# The prognosis of delirium in older outpatients

**DOI:** 10.1111/psyg.13078

**Published:** 2024-01-23

**Authors:** Daisy WP Quispel‐Aggenbach, Sytse U Zuidema, Hendrika J Luijendijk

**Affiliations:** ^1^ Department of Primary and Long‐term Care University of Groningen, University Medical Center Groningen Groningen The Netherlands; ^2^ Department of Elderly Psychiatry Parnassia Rotterdam The Netherlands

**Keywords:** delirium, elderly, older patient, prevalence, prognosis, risk factors

## Abstract

**Background:**

Delirium is a common and serious neuropsychiatric disorder. The prognosis of delirium in older patients living at home has not been studied often before. The aim of this study is to examine the prognosis of delirium in patients attending a memory clinic of a psychiatric hospital.

**Method:**

The study population consisted of 85 outpatients diagnosed with delirium between October 2013 and October 2014. Seventeen patients had already been diagnosed as having dementia. Three months after the diagnosis, consenting patients underwent a follow‐up visit. We recorded delirium status (remitted or not), new dementia diagnosis, subjective cognitive functioning compared to baseline and to before delirium, level of daily functioning, and place of residence.

**Results:**

After 3 months, 45 (53%) had recovered from delirium, 19 (22,4%) had persistent/recurrent delirium, 12 (14%) patients had died, and another nine (11%) could not be revisited for other reasons than death. None of the 64 re‐examined patients reported that their cognitive functioning had recovered to the pre‐delirium level, and the mean level of daily functioning did not substantially improve either. The rate of diagnosed dementia increased to 63.8%, and 18 patients (28.1%) had moved to a nursing home.

**Conclusions:**

Delirium in older outpatients has a poor prognosis. A larger study on the risk factors of the prognosis of delirium in older persons living at home is advised.

## INTRODUCTION

Delirium is a common and serious neuropsychiatric disorder. The key symptom is an acute and fluctuating attention disorder, which is often accompanied by disorders of cognitive function, orientation, thought processing, perception, and sleep–wake cycle.^1^ Common triggers are infection, drug intoxication, drug withdrawal, metabolic disturbances and endocrine disturbances. Older age, prior delirium and dementia are non‐modifiable risk factors for delirium.^2^, ^3^ Delirium occurs in 10% to 40% of hospitalised and institutionalised patients.^2^, ^4^ Studies among older outpatients referred for dementia screening to psychiatric outpatient clinics have reported a prevalence of delirium between 16% and 19%.^1^, ^3^, ^5^

The prognosis of delirium is poor in hospitalised and institutionalised patients. A review of 18 studies reported that it was still present among 45% of hospitalised patients at discharge and among 21% after 6 months.^6^ Also, delirium may have potentially severe consequences such as poor cognitive and functional recovery, longer hospital stay, increased risk of nursing home placement, and death.^6^, ^7^, ^8^ A review of 14 studies in residents of nursing homes also showed that delirium is often persistent and associated with increased risk of mortality.^2^ In one study, delirium did not resolve completely in 67% of the patients, and 24% of patients died in the first month after delirium onset.^9^

Just a few studies have investigated the prognosis of delirium in older patients at home. In the population‐based Finish Vantaa 85+ study, 199 non‐demented participants were re‐examined after 3 years.^10^ Twenty (10%) had experienced one or more episodes of delirium since baseline. At the time of re‐assessment, 65% of them had dementia, and 55% died in the 2 years following the re‐assessment. In another population‐based study among 3330 Canadians, 21 non‐demented persons had delirium at the baseline or at a 5‐year follow‐up assessment. Of them, 82% died during the 5 years following the diagnosis.^11^ No other outcomes were reported. Both studies were based on a small number of patients with delirium, and also included nursing home residents, and none of them had a history of dementia. More recently, a study among 109 outpatients diagnosed with delirium in a memory clinic was published.^12^ All patients had dementia or mild cognitive impairment. At 6 months of follow‐up, 29% had died, and 28% had moved to a nursing home.

There is a scarcity of studies about the prognosis of delirium in community‐dwelling persons with and without dementia. The aim of this study was to examine the course of delirium and its determinants in older outpatients of a memory clinic.

## MATERIALS AND METHODS

### Design and participants

The current study is an extension of a prior study about the prevalence of delirium. That study was performed among 444 older patients consecutively referred to an outpatient clinic of a psychiatric institution between March 2013 and March 2014.^3^ They lived in or close to Rotterdam, the Netherlands. A general practitioner or geriatrician had referred them for dementia screening. They had cognitive disorders with or without psychological or behavioural disorders, and some were suspected of having delirium at the time of referral. In the prevalence study, 85 of 444 patients were diagnosed with probable delirium at the time of intake. For the current study, the 85 patients with delirium formed the study population.

The study protocol was approved by the Medical Ethics Committee of the Erasmus University of Rotterdam, The Netherlands. The committee granted a waiver for consent of patients, because data were collected as part of (enhanced) medical care. The study did not pose a risk to the patients. The Strengthening the Reporting of Observational Studies in Epidemiology Guidelines were used to present our work.^13^

### Baseline assessment

At baseline, the patients were visited twice (if necessary, more often), once by a registered (psychiatric) nurse or psychologist, and once by a geriatrician or psychiatrist. The patient and caregiver were interviewed about the presence of delirium symptoms, and fluctuations in cognitive, psychological and behavioural functioning during and across days. Afterwards, the interviewer filled out the Delirium Rating Scale‐Revised‐98 (DRS‐R‐98).^14^ The first 13 items of this scale cover the presence and severity of delirium symptoms, and the last three items consist of diagnostic criteria (acute onset, fluctuations and somatic illness). In addition, the use of medication and changes therein shortly before the onset of delirium symptoms were registered. A physical exam was performed, and blood and urine tests were ordered. If necessary, the medical file was checked, and additional information about underlying somatic conditions obtained from the general practitioner or hospital‐based specialist.

The diagnosis of probable delirium was based on the criteria for delirium outlined in the Diagnostic and Statistical Manual of Mental Disorders, 4th Edition, Text revision (DSM‐IV‐TR). If one criterion was not met, possible delirium was registered. We recorded which somatic factors could have triggered or contributed to the occurrence of delirium: drugs, metabolic/endocrine disorder, infection, neoplasm, cerebrovascular attack, heart failure, and other illnesses like head trauma, pain, constipation or major surgery.

If cognitive decline was present before onset of delirium but no formal diagnosis of dementia had been made yet, the diagnosis of dementia was postponed.

In addition, we registered age, sex and place of residence. Finally, we assessed functionally impaired hearing (absent/slight limitation/present), impaired sight (absent/slight limitation/present), walking inside (unable/able), and level of activities of daily living (ADL) on the Katz‐scale (sum of items, range 0–6, higher is worse).

### Follow‐up visit

The patients with probable delirium at baseline were visited again after 3 months of follow‐up. The patient and caregiver were interviewed about the presence of delirium symptoms, fluctuations during and across days, and course of the syndrome since baseline. Severity of delirium was scored with the DRS‐R‐98, and a diagnosis of remitted or recurrent/persistent delirium was made. We asked the patient and caregiver to score the level of cognitive functioning on a three‐point scale as the same, better, or worse compared to baseline and to before delirium based on their own impression. Again, place of residence was registered, and level of ADL.

In some patients, delirium had remitted but cognitive dysfunction was still present, or delirium had not remitted but cognitive impairment had been present for some time before the onset of delirium. We obtained more information about type, severity, onset, and course of the cognitive impairment from these patients and their caregivers. We also administered the Mini‐Mental State Examination, and clock drawing test. A diagnosis of dementia was made according to DSM‐IV‐TR criteria.

### Statistical analysis

First, we used descriptive statistics to describe the study population at baseline. We calculated the means with standard deviations (SD) for continuous data, and proportion for binary data.

Second, we calculated the percentage of patients with the following outcomes after 3 months: (i) remitted delirium; (ii) recurrent/persistent delirium; and (iii) death. We then described the 3 months characteristics of the first two groups with means (SD) for continuous data, and proportions for binary data. We used the independent sample *t*‐test for means and Chi‐square test for binary data to determine whether characteristics differed between the patients with remitted and recurrent/persistent delirium.

Finally, we used logistic regression to investigate risk factors of death or recurrent/persistent delirium at follow‐up. The investigated risk factors were age above 90 years, sex, history of diagnosed dementia at baseline, impaired ADL, impaired hearing, impaired sight, polypharmacy, recent hospitalisation, and triggers of delirium (drugs, metabolic/endocrine disorder, infection, neoplasm, cerebrovascular attack, heart failure, and other illnesses). Each analysis of the association between an individual risk factor and an outcome was adjusted for age (continuous) and sex. We analyzed the data with Stata 17.0.^15^

## RESULTS

### Study population

Table 1 shows the characteristics of the study population (*N* = 85). The mean age of the patients was 86.6 (SD 7.5), and 63 (74%) were female. Sixty‐one patients (72%) lived at home and the others in a care centre. Fifteen patients (18%) had a history of delirium. Prior to the delirium diagnosed at baseline, six patients (7%) were diagnosed with dementia and 11 (13%) with other cognitive disorders. The mean level of daily activities was 3.0 (SD 1.5) on the Katz‐scale.

**Table 1:** Characteristics of study population

	Patients with probable delirium (*N* = 85)
Demographic characteristics
Age, mean (SD)	86.6 (7.5)
Sex, *n* (%)	
Male	22 (25.9)
Female	63 (74.1)
Place of residence, *n* (%)	
Home	61 (71.8)
Care centre	24 (28.2)
Medical history	
History of delirium, *n* (%)	15 (17.7)
History of dementia^†^, *n* (%)	
No cognitive disorder	68 (80.0)
Other cognitive disorders	11 (12.9)
Dementia, any type	6 (7.1)
Number of drugs	
Mean (SD)	7.1 (4.0)
5 or more medicines, *n* (%)	63 (74.1)
Recent hospital discharge, *n* (%)	27 (31.8)
ADL, mean (SD)	3.0 (1.5)
Impaired hearing, *n* (%)	34 (40.0)
Impaired sight, *n* (%)	26 (30.6)
Baseline delirium	
DRS‐R‐98 severity score, mean (SD)^‡^	14.3 (6.1)
DRS‐R‐98 total score, mean (SD)^§^	18.5 (7.2)
Drugs, *n* (%)	82 (96.5)
Probable trigger	19 (22.4)
Metabolic/endocrine disorder, *n* (%)	46 (54.1)
Probable trigger	10 (11.8)
Infection, *n* (%)	40 (47.1)
Probable trigger	36 (42.4)
Neoplasm, *n* (%)	6 (7.1)
Probable trigger	4 (4.7)
Cerebrovascular attack, *n* (%)	20 (23.5)
Probable trigger	2 (2.4)
Heart failure, *n* (%)	20 (23.5)
Probable trigger	5 (5.9)
Other illnesses, *n* (%)	20 (23.5)
Probable trigger	4 (4.7)

*Note*: ADL, activities of daily living; DRS‐R‐98, Delirium Rating Scale‐Revised 1998; SD, standard deviation.

^†^ Dementia diagnosis before intake.

‡ Items 1–13.

^§^ Items 1–16.

The trigger of delirium was most often an infection, a drug intoxication or – withdrawal, or a metabolic/endocrine disturbance. Many patients had more than one trigger. In most cases, it was possible to treat the trigger(s) successfully.

### Prognosis

**Figure 1:**
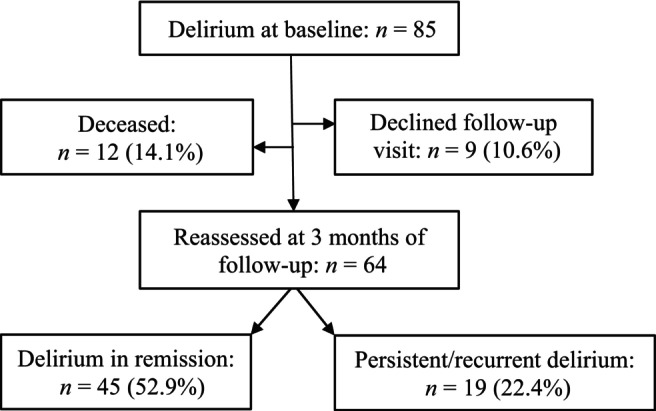
Flow diagram of participants.

Figure 1 shows the outcome after 3 months of follow‐up. Twenty‐one patients could not be revisited, because 12 patients had died (14.1%), and nine patients did not give their permission (10.6%), for example, because they were too ill (*n* = 4). Sixty‐four of 85 patients were interviewed. Delirium had remitted in 45 patients (52.9%), and it had recurred or was persistent in 19 patients (22.4%).

Table 2 shows the 3 months characteristics of the groups with remitted and recurrent/persistent delirium. Eighteen patients had moved to a nursing home (28.1%), but patients with recurrent/persistent delirium did not have more ADL impairments on average, or an increased chance of institutionalisation.

**Table 2:** Characteristics of patients revisited after 3 months of follow‐up

	Delirium in remission (*n* = 45)	Recurrent or persistent delirium (*n* = 19)
Demographic characteristics
Age, mean (SD)	86.1 (7.2)	86.7 (7.9)
Sex, *n* (%)		
Male	12 (26.7)	5 (26.3)
Female	33 (73.3)	14 (73.7)
Place of residence, *n* (%)		
Home	20 (44.4)	9 (47.4)
Care centre	13 (28.9)	4 (21.1)
Nursing home	12 (26.7)	6 (31.6)
Medical status		
Dementia diagnosis, *n* (%)^†^		
No cognitive disorder	6 (13.3)	5 (26.3)
Other cognitive disorders	13 (28.9)	6 (31.6)
Dementia, any type	26 (57.8)	8 (42.1)
ADL, mean (SD)	3.0 (1.1)	3.2 (1.7)
Impaired hearing, *n* (%)	19 (42.2)	6 (31.6)
Impaired sight, *n* (%)	13 (28.9)	7 (36.8)
Cognitive function compared to baseline (*n*, %): better	36 (80.0)	11 (57.9)^‡^
Similar	4 (8.9)	7 (36.8)^‡^
Worse	5 (11.1)	1 (5.3)^‡^
Cognitive function compared to before delirium (*n*, %): better	0 (0)	0 (0)^§^
Similar	12 (26.7)	1 (5.3)^§^
Worse	33 (73.3)	18 (94.7)^§^

*Note*: ADL, activities of daily living, SD, standard deviation.

^†^ Diagnosis at 3 months follow‐up visit.

^‡^ *P* = 0.024.

^§^ *P* = 0.052.

Self‐reported cognitive functioning had improved compared to baseline in 36 of 45 patients with remitted delirium (80%) and 11 of 19 patients with recurrent/persistent delirium (58%). No patient reported that cognitive functioning had improved to the level before the delirium. In addition, dementia or another cognitive disorder was diagnosed during the follow‐up visit in 39 of 45 patients with remitted delirium (86.6%), and 14 of 19 patients (73.7%) with recurrent/persistent delirium.

### Risk factors

Table 3 shows the association between characteristics at baseline and death at 3 months of follow‐up. Age >90 years, impaired ADL, hospital discharge within 3 months before baseline, and cerebrovascular attack as a trigger of delirium seemed to increase the risk of death. However, the estimated risks were uncertain due to large confidence intervals.

**Table 3:** Association of risk factors at baseline with death or recurrent/persistent delirium at 3 months of follow‐up in patients with delirium at baseline

	Death, OR (95% CI); *N* = 85^†^	Recurrent/persistent delirium, OR (95% CI); *N* = 64^†^
Demographic characteristics		
Age >90 years	2.72 (0.74*–*9.96)	1.49 (0.50–4.45)
Female sex	1.61 (0.31–8.28)	0.99 (0.29–3.39)
Medical history		
Dementia/cognitive disorder	0.78 (0.15–3.98)	1.06 (0.28–3.99)
Impaired ADL^‡^	4.50 (0.49–41.2)	1.37 (0.39–4.84)
Impaired hearing	0.86 (0.23–3.16)	0.57 (0.17–1.88)
Impaired sight	0.31 (0.06–1.61)	1.68 (0.47–6.01)
Polypharmacy^§^	1.81 (0.36–9.11)	2.17 (0.54–8.73)
Hospital discharge in last 3 months	2.10 (0.56–7.96)	0.37 (0.10–1.38)
Triggers of delirium		
Drug intoxication/withdrawal	1.70 (0.37–7.79)	1.86 (0.51–6.73)
Metabolic/endocrine disorders	0.60 (0.07–5.34)	0.30 (0.04–2.64)
Infection	0.56 (0.15–2.06)	1.00 (0.33–3.04)
Neoplasm	NE	0.76 (0.07–8.55)
Cerebrovascular attack	4.92 (0.27–88.7)	NE
Heart failure	NE	0.52 (0.05–5.36)
Other illnesses	NE	2.64 (0.33–20.9)

*Note*: ADL, activities of daily living; CI, confidence interval; NE, not estimable (due to one or more zero cells); OR, odds ratio.

^†^ Corrected for age (continuous) and sex.

^‡^ Three or more on Katz‐scale.

^§^ Five or more drugs.

In addition, Table 3 shows the relationship of risk factors at baseline with recurrent/persistent delirium at the 3 months visit. Polypharmacy and other illnesses as triggers for delirium may increase the risk of recurrent/persistent delirium, but again, confidence intervals were wide.

## DISCUSSION

We examined the 3 months prognosis of delirium in 85 older outpatients. It was generally poor. Twelve patients died within 3 months, and 19 of 64 patients interviewed after 3 months had ongoing delirium. Nevertheless, 45 recovered from delirium, and statistically significantly more of them reported an improved cognitive functioning compared to the time of diagnosis. However, no patient reported that cognitive functioning recovered to the pre‐delirium level, and the average ADL level remained the same. Eighteen patients had moved to a nursing home.

### Prognosis and its risk factors

We found that 14% of the older outpatients with delirium died within 3 months of the diagnosis. This is a high rate, but more favourable than for hospitalised and institutionalised delirious patients. A review of hospital studies reported a probable mortality rate at discharge of 15% to 37%.^16^ In nursing home patients, one study found a mortality rate of 24% at 1 month, and another study a mortality rate of 34% at 3 months.^9^, ^17^ Two previous studies in small community‐dwelling non‐demented populations also found that delirium increased the risk of death. In a Finish study, 55% of participants with delirium died during the 2 years of follow‐up compared to 26% of participants without delirium.^10^ In a Canadian study, 82% of persons with delirium died during the 5 years following the diagnosis compared to 30% of persons without delirium.^11^

We also found that 22,4% of the patients (19 patients) still had delirium after 3 months of follow‐up. This finding concurs with that of studies in older hospitalised patients. A review of such studies showed that delirium was persistent in 27% of patients after 3 months.^6^ A prolonged or chronic delirium has many similarities with frailty and may be associated with negative outcomes.^18^, ^19^ A study among post‐acute care patients found that subjects with persistent delirium were 2.9 times more likely to die during the 1‐year follow‐up period compared to subjects with resolved delirium.^18^ This association was present in groups with and without dementia.^18^

Although the risk estimate is uncertain, polypharmacy may increase the risk of recurrent/persistent delirium according to our results. As this is a modifiable risk factor, this finding should be further investigated. A review of 21 studies reported that dementia, increasing numbers of medical conditions, increasing severity of delirium, hypoactive symptoms and hypoxic illnesses were associated with persistent delirium.^20^

In our study, more than half of the patients with delirium could be treated effectively and the delirium remitted. This is higher than the 33% reported in hospitalised patients.^21^ Also, 80% of patients with remitted delirium reported a recovery of cognitive function. Yet, no patients reported that their cognitive functioning had improved to the pre‐delirium level. In addition, the rate of new diagnoses of dementia at 3 months of follow‐up was high: at baseline, 17 of 85 (20.0%) patients had diagnosed dementia or another cognitive disorder, and the rate increased to 53 of 64 (63.8%) in the re‐examined patients. Also, more than a quarter of the study population had moved to a nursing home, and this rate was not statistically different for patients with remitted delirium compared to recurrent/persistent delirium. This finding seems to confirm the clinical experience that delirium is often a sign of pre‐existent dementia.^7^ ^,^ ^22^

Adequate detection of delirium is a prerequisite for effective treatment, but it is often missed, especially in patients with pre‐existent cognitive impairment. Early detection might also help to prevent negative outcomes of delirium, such as prolonged delirium and related cognitive impairment. During our study, physicians and psychiatric nurses who were familiar with performing psychiatric examinations could implement the DRS‐R‐98 fairly easily, because it helped to structure the diagnostic work‐up without taking a significant extra effort. Another advantage of this scale is that it is not administered to the patient. We believe that the use of this validated diagnostic tool helped our team to detect delirium more often. In addition, we developed a short delirium caregiver questionnaire that helped to detect delirium quicker as well in our population of older outpatients with cognitive impairment.^23^

### Strengths and limitations

This is one of the first studies that examined the prognosis of delirium in older outpatients. The data was collected by experienced physicians and psychiatric nurses using a validated diagnostic tool for delirium. We also explored risk factors of a poor course of delirium. A limitation was the small sample, which resulted in uncertain estimates concerning the risk factors of death and persistent/recurrent delirium after 3 months of follow‐up. Larger studies on delirium in elderly persons living at home are needed. In a new study, it would be useful to add the results after 3 months from a control group that has not experienced delirium so that the effect of going through delirium can be even more precisely determined.

### CONCLUSION

Our study showed that half of patients could be treated effectively and delirium remitted. Nevertheless, the other patients had a poor prognosis. They died within 3 months, or the delirium persisted and developed into a chronic state.

## DISCLOSURE

The authors have no conflicts of interest to declare.

## Data Availability

The data that support the findings of this study are available on request from the corresponding author. The data are not publicly available due to privacy or ethical restrictions.
